# Association of gait speed and grip strength with risk of cardiovascular events in patients on haemodialysis: a prospective study

**DOI:** 10.1186/s12882-019-1370-6

**Published:** 2019-05-30

**Authors:** Atsumi Kuki, Kentaro Tanaka, Akifumi Kushiyama, Yoshihide Tanaka, Shuta Motonishi, Yasuji Sugano, Toru Furuya, Takashi Ozawa

**Affiliations:** 1 Kitahachiouji Clinic, 2960-5 Ishikawa-cho, Hochiuji-shi, Tokyo, 1920032 Japan; 2 Higashikurume Ekimae Clinic, 1-3-6 Honmachi, Higashikuruume-shi, Tokyo, 2030053 Japan; 3 The Division of Diabetes and Metabolism, The Institute for Adult Diseases, Asahi Life Foundation, 2-2-6, Bakuro-cho, Chuo-ku, Tokyo, 103-0002 Japan; 4 Department of Pharmacotherapy, Meiji Pharmaceutical University, 2-522-1 Noshio, Kiyose, Tokyo, 204-8588 Japan; 5 Kumegawa tousekinaika Clinic, 3-6-3 Onda-cho, Musashimurayama-shi, Tokyo, 189-0011 Japan; 6 Higashiyamato Nangai Clinic, 4-2-8 Nangai, Higashiyamato-shi, Tokyo, 2070014 Japan; 7 Higashikurume Clinic, 2-2-22 Shinkawa-cho, Higashikurume-shi, Tokyo, 2030013 Japan; 8 Kodaira Kitaguchi Clinic, 2-2-11 Onuma-cho, Kodaira-shi, Tokyo, 1870001 Japan

**Keywords:** Chronic haemodialysis, Physical activity, Prognosis, Cardiovascular events

## Abstract

**Background:**

Gait speed (GS) and handgrip strength (HGS), both factors associated with frailty and sarcopenia, are reportedly associated with CV events in the general population. However, little is known about the impact of these factors on the outcome of patients on dialysis. This study aimed to evaluate whether evaluation of GS and HGS could be associated the onset of fatal/non-fatal cardiovascular (CV) events in patients on haemodialysis (HD).

**Methods:**

One-hundred-eighty-two patients with end-stage renal disease (ESRD) undergoing HD at four dialysis clinics in April 2015 provided written informed consent to participate in the study. We excluded patients who had physical disability, were unable to walk without help, or had recently experienced CV events. Usual GS over a 4-m walk and HGS were measured at baseline, and 173 patients (men, 124; women, 49) were divided into sex-specific quartiles according to GS and HGS and were followed-up for fatal/non-fatal CV events for a median of 2 years. We examined the association of GS and HGS with CV events and determined cut-off values using Cox regression analysis adjusted for age, sex, HD duration, history of CVD, and diabetes.

**Results:**

During the follow-up period, 46 CV events occurred. Both physical performance factors were significantly associated with CV events. Low GS (< 0.82 m/s for men and 0.81 m/s for women) and weak HGS (< 29.0 kg for men and 19.7 kg for women) were associated with CV events. For low vs. high GS, the hazard ratio (HR) for CV events was 2.29 [95% confidence interval (CI): 1.20–4.33; *P* = 0.01], and for low vs. high HGS, the HR was 2.15 [95% CI: 1.00–5.04; *P* < 0.05]. These HRs remained significant after adjusting for confounding factors, such as sex, age, dialysis vintage, history of CV disease, and diabetes.

**Conclusions:**

Slow GS and weak HGS in patients on HD were suggested to be independent predictors of fatal/non-fatal CV events.

**Electronic supplementary material:**

The online version of this article (10.1186/s12882-019-1370-6) contains supplementary material, which is available to authorized users.

## Background

With the increase in the ageing Japanese population, there is an increase in the number of aging patients with end stage renal disease (ESRD) [1]. The average age of Japanese dialysis patients was 51.3 years in 1983, which reached 69.4 years by the end of 2016 [1]. Frailty and sarcopenia are highly prevalent in elderly patients with ESRD undergoing dialysis [2–4]. The deterioration of renal function leads to a variety of metabolic disorders in patients with ESRD, including chronic inflammation, uraemia, oxidative stress, insulin resistance, and malnutrition, which increases the risk of frailty and sarcopenia [5, 6].

Frailty and sarcopenia were associated with poor outcomes, including not only falls, incident disability, hospitalization, and mortality [7, 8], but also cardiovascular (CV) events [9], such as myocardial infarction (MI), heart failure, and cerebrovascular disorders. The frailty assessment includes the following five items in the frailty phenotype protocol: weight loss, exhaustion, low physical activity level, low handgrip strength (HGS) as weakness, and slow gait speed (GS) as physical disability [10]. Sarcopenia is mainly characterized by atrophy of skeletal muscles, along with a decrease in muscle strength and function and exhibits physical function impairment—a common aspect of frailty [11]. Previous studies have suggested that several features of frailty or sarcopenia, such as exhaustion, slow GS, low HGS, low physical activity level, and low skeletal muscle mass, constitute the criteria associated with the onset of CV events [12, 13].

GS and HGS are the common physical parameters associated with frailty and sarcopenia. These two parameters have been reported to be potential prognostic indicators of CV events in the general elderly population [12]. According to a previous systematic review, GS is significantly associated with cardiovascular risk factors (hypertension, diabetes, and intima-media thickness) and CV events (peripheral artery disease [PAD], stroke, and CV mortality) in the older population [14]. HGS is a well-established indicator of overall muscle strength. A previous meta-analysis reported a linear association between HGS and CV events in a community-dwelling population [15]. Furthermore, HGS was found to be inversely associated with CV events in a study including 1.1 million young Swedish men [16].

In patients undergoing HD, CV events are the leading cause of death [17], however, little information is available with regards to quantitative risk assessment for CV events by frailty and sarcopenia. Therefore, this study aimed to prospectively evaluate whether the two physical performance parameters are associated with the onset of fatal/non-fatal CV events in patients undergoing HD. In particular, we also examined whether there were thresholds in the two measurements., with certain threshold.

## Methods

### Trial design

The present study was a 2-year prospective observational cohort study to examine the association between GS/HGS and CV events in patients with ESRD undergoing HD at multiple clinics. The protocol was approved by the Institutional Ethics Committee of Medical Toyou, Japan, and we obtained written informed consent from all participants after the protocol was explained in detail. This study was conducted in accordance with the ethical standards of the responsible committee on human experimentation and with the principles of the Helsinki Declaration.

### Study population

We recruited 182 outpatients with chronic kidney disease (CKD) stage-5 undergoing HD at our four outpatient dialysis clinics (Kitahachiouji Clinic, Higasikurume Clinic, Higashiyamato Nangai Clinic, and Kodairakitaguchi Clinic) in Tokyo, Japan, from January 2015 to March 2015.

**Fig. 1:**
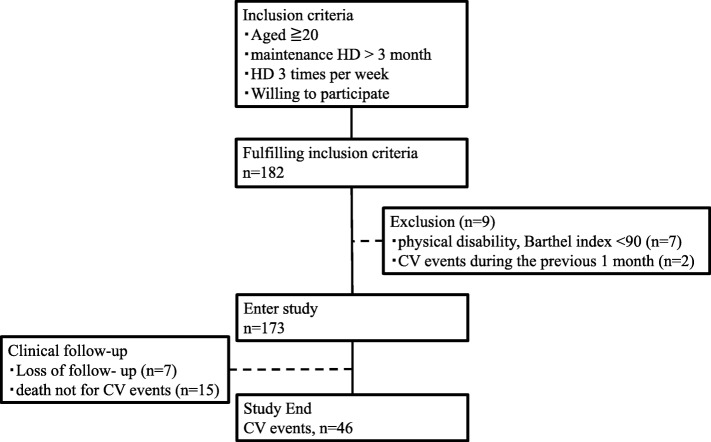
Patient recruitment flow diagram, study selection process and follow-up

Eligibility criteria included age ≥ 20 years at enrolment and undergoing HD three times per week for more than 3 months. Patients were excluded if they exhibited physical disability (Barthel index < 90) [18] or had a history of CV events within 1 month before the measurement of GS and HGS. We excluded nine patients with physical disability (*n* = 7), and with a recent history of CV events (*n* = 2). The remaining 173 patients (124 men and 49 women, age 34–91 years) were enrolled in the study (Fig. 1).

### Data collection

Demographic and clinical data, including age, sex, and comorbidities were collected by retrieving medical records and asking the patients directly. Laboratory parameters and dialysis adequacy were measured before the first HD session of the week. The single-pool Kt/V was used to determine the weekly dialysis dose. Subjects with diabetes were defined as those who had a previous diagnosis of diabetes by a physician or had a haemoglobin A1c (HbA1c) level > 6.5%. History of CVD included history of medical therapy or surgical treatment for MI, angina, PAD, and hospitalization for congestive heart failure or stroke.

### Physical performance parameters

Physical performance was assessed at the baseline. A walking test was performed along a 4-m walkway with a 1-m start-up from the starting point at individual patients’ usual pace. All participants started to walk from the standing position. GS (m/s) was calculated by dividing the distance covered by time (s) [19].

HGS was measured using a hand-held dynamometer (Takei Scientific Instruments Co, Niigata, Japan). After adjusting the dynamometer for hand comfort, the HGS (in kg) was measured in a sitting position with the arm hanging by the side and the elbow fully extended. The highest grip strength after maximal efforts was recorded for each hand twice. For the present analysis, we used the maximum values obtained from both hands. Both usual gait speed and grip force measurements were performed before the dialysis session on either the first, second, or third dialysis day of the week.

### Ascertainment of cardiovascular events

The primary endpoint was incident CV events during the observational period from 1 April 2015 to 1 April 2017. Patients were reviewed during a median 2-year follow-up period by medical records and information from interviews. Follow-up was performed from enrolment until the first event, or loss to follow-up due to any reason, such as transfer to another clinic, whichever occurred first. CV events were defined as CV death, MI, stroke, angina, hospitalizations for congestive heart failure, and PAD.

MI and angina were confirmed using coronary angiography or myocardial scintigraphy. Stroke was defined as haemorrhagic or ischaemic stroke, which included lacunar infarctions with symptoms confirmed by brain computed tomography (CT) or magnetic resonance imaging (MRI). PAD was diagnosed using angiography with enhanced CT or MRI. Only one first event per subject was included in the analysis. The onset of CV events without sudden death was defined as the medical or surgical treatment initiation date.

### Statistical analysis

Subjects were divided into sex-specific quartiles, ranging from Q1 (the lowest quartile) to Q4 (the highest quartile), according to baseline GS and HGS. The cut-offs were as follows: GS (m/s): < 0.82, 0.82–0.95, 0.95–1.15, and > 1.15 for men, and < 0.81, 0.81–0.93, 0.93–1.11, and > 1.11 for women; HGS (kg): < 22.1, 22.1–26.2, 26.2–32.1, and > 32.1 for men, and < 15.0, 15.0–17.1, 17.1–21.6, and > 21.6 for women.

Demographic data were summarized using the mean and standard deviation or median with 25th and 75th percentiles for continuous variables, as appropriate; and count and proportion for categorical data. Spearman’s correlation analysis was used to analyse the association between GS and HGS. Comparison of baseline characteristics between the GS and HGS quartiles was performed using the Kruskal–Wallis test and Cochran–Armitage trend test for continuous and categorical variables, respectively. The incidence rates of CV events were estimated via the Kaplan–Meyer method and compared using a log-rank test according to GS and HGS categories.

Cox proportional hazards models were used to evaluate the association between GS and HGS and CV events. To evaluate the threshold, GS and HGS were divided into 16 sex-specific quantiles. At each quantile point (6.25th percentile), we made two groups—below and above the quantile—and calculated the hazard ratios (HRs) and 95% confidence intervals (CIs) for the low vs. high GS and HGS groups. Multivariate Cox regression analyses were performed to determine independent prognostic effect of GS and HGS, divided according to the obtained threshold values for CV events, after adjusting for the following confounders: age, sex, HD duration, history of CVD, and diabetes. All analyses were performed using JMP software (version 13.0; SAS Institute, Cary, NC, USA). Statistical significance was set at *P* < 0.05.

## Results

In the present study, 173 patients with ESRD (124 men and 49 women) undergoing HD were enrolled. Table 1 shows the baseline clinical characteristics of all patients and subgroups according to GS and HGS quartiles. The median (interquartile-range [IQR]) age was 69.0 years (62.5–76.0 years), and 71.7% of participants were male. The patients’ median (IQR) HD duration was 5.5 years (2.4–9.9 years). Fifty percent of the patients were diabetic, and 42.8% had a history of CVD. The mean GS was 1.0 ± 0.3 m/s for men and 0.9 ± 0.2 m/s for women, and the mean HGS was 27.2 ± 7.1 kg for men and 17.7 ± 4.6 kg for women. GS correlated with HGS in both men (ρ = 0.49, *P* < 0.01) and women (ρ = 0.36, *P* = 0.03) by Spearman’s correlation analysis.

**Table 1:** Baseline clinical and biochemical characteristics of HD patients according to sex-specific quartiles of gait speed and handgrip strength

Characteristics	Total	Normal gait speed					Handgrip strength				
		Q1 (*n*=43)	Q2 (*n*=42)	Q3 (*n*=45)	Q4 (*n*=43)	*p* value	Q1 (*n*=43)	Q2 (*n*=43)	Q3 (*n*=39)	Q4 (*n*=48)	*p* value
Sex (men/women)	124/49	31/12	31/12	31/14	31/12	0.87	31/12	31/12	30/9	32/16	0.66
Age (year)	69.0 (62.5-76.0)	72.5±9.7	70.8±10.1	64.4±11.1	63.9±12.3	<0.01	72.5±9.7	70.0 (66.0-76.0)	67.0 (63.0-73.0)	65.5 (52.0-69.8)	<0.01
Duration of dialysis (year)	5.5 (2.4-9.9)	6.9±6.8	6.7±5.9	5.4±3.9	9.7±7.9	0.06	6.9±6.8	4.5 (1.7-11.1)	3.6 (1.6-6.7)	6.0 (2.9-10.6)	0.02
Body mass index (kg/m2)	21.7 (19.4-24.7)	22.0±4.1	23.0±3.3	23.1±4.7	21.0±3.6	0.03	22.0±4.1	22.1 (19.3-25.3)	22.4 (20.6-26.0)	21.8 (19.6-24.8)	<0.01
Albumin (g/dl)	3.8 (3.6-3.9)	3.7 (3.5-3.8)	3.8 (3.5-4.0)	3.8 (3.6-3.9)	3.8 (3.6-4.0)	0.19	3.7 (3.5-3.8)	3.7 (3.6-3.9)	3.7 (3.6-3.9)	3.8 (3.7-4.0)	<0.01
HDL-cholesterol (mg/dl)	41.0 (33.5-51.0)	41.0 (33.5-50.0)	41.0 (32.0-53.3)	39.0 (31.0-46.0)	49.0 (39.0-54.0)	0.19	41.0 (33.0-49.0)	43.0 (33.0-55.0)	39.0 (31.0-48.0)	41.0 (33-49)	0.48
LDL-cholesterol (mg/dl)	77.0 (62.5-98)	71.1±23.7	81.4±24.5	81.8±26.9	83.0±27.7	0.07	78.8±19.3	79.7±28.0	80.6±29.9	81.5±26.6	0.96
C-reactive protein (mg/dl)	0.1 (0.05-0.3)	0.1 (0.06-0.3)	0.1 (0.05-0.5)	0.2 (0.05-0.3)	0.06 (0.05-0.3)	0.28	0.1 (0.05-0.5)	0.2 (0.05-0.3)	0.1 (0.05-0.6)	0.06 (0.05-0.2)	0.24
Creatinine (mg/dl)	10.8±2.5	9.5±2.5	11.0±2.7	11.2±2.4	11.42.0	<0.01	9.8±1.5	10.2±2.6	11.4±2.9	11.7±2.5	<0.01
Ca^a^ (mg/dl)	9.0±0.6	9.0±0.7	9.2±0.7	8.9±0.5	8.90.7	0.11	9.1±0.6	8.8±0.6	9.2±0.8	8.9±0.6	0.03
P (mg/dl)	5.4±1.2	4.9±1.3	5.4±1.3	5.4±1.1	5.7±1.1	0.05	5.1±1.2	5.2±1.2	5.6±1.1	5.6±1.2	0.25
intact PTH (pg/ml)	133.0 (75.0-190.5)	108.0 (52.0-141.0)	124.0 (68.8-186.3)	154.0 (89.5-263.0)	151.0 (107.0-225.0)	10	127.0 (54.0-169.0)	122.0 (76-176)	137.0 (93-187)	152.5 (88.0-227.0)	0.33
β2MG (mg/L)	26.4±5.6	26.2±6.7	26.5±5.0	26.0±5.5	26.9±4.9	0.9	27.1±5.1	26.7±7.0	26.3±4.8	25.6±5.1	0.38
%CGR	92.0±22.4	88.6±18.3	84.7±18.3	88.0±24.0	95.4±18.8	0.06	88.6±18.3	88.0±24.0	95.8±27.7	95.4±18.8	0.14
Single-pool Kt/V	1.4 (1.3-1.6)	1.5±0.3	1.4±0.2	1.5±0.2	1.60±.2	0.01	1.5±0.2	1.5±0.2	1.4±0.2	1.5±0.3	0.35
Comorbid condition											
Diabetes (n, (%))	86 (50.0)	27(62.8)	25(60.0)	22(48.9)	12(27.9)	<0.01	24 (55.8)	24 (55.8)	20 (51.3)	17(35.4)	0.07
History of CVD^b^ (n, (%))	74 (42.8)	21(48.8)	21 (48.8)	20 (46.5)	17 (35.4)	<0.01	21(48.8)	20 (45.6)	17 (43.6)	17 (35.4)	0.18
Heart failure^c^ (n, (%))	6 (3.5)	0 (0)	1 (2.4)	2 (4.4)	3 (7.0)	0.07	1 (2.3)	2 (4.7)	1 (2.6)	2 (4.2)	0.77
Myocardial infarction and angina (n, (%))	31 (17.9)	10 (23.3)	9 (21.4)	7 (15.6)	5 (11.6)	0.11	9 (21.0)	8 (18.6)	8 (20.5)	6 (17.9)	0.34
Peripheral arterial disease, (n, (%))	18 (10.4)	11(25.6)	4 (9.5)	2 (4.4)	1 (2.3)	<0.01	9 (20.9)	4 (9.3)	5 (12.8)	0 (0.0)	<0.01
Stroke (n, (%))	36 (20.8)	14 (32.6)	11(26.2)	7 (15.6)	4 (9.3)	< 0.01	12 (27.9)	10 (23.3)	7 (18.0)	7 (14.6)	0.09
Dementia (n, (%))	9 (5.2)	6 (14.0)	1 (2.4)	1 (2.2)	1 (2.3)	0.02	5 (11.6)	3 (7.0)	0 (2.1)	1 (5.2)	0.02
Hemiplegia (n, (%))	17 (9.8)	8 (18.6)	6 (14.3)	2 (4.4)	1 (2.3)	<0.01	5 (11.6)	3 (7.0)	6 (15.4)	3 (6.3)	0.64
Presence of malignancy^d^ (n,(%))	7 (4.0)	3 (7.0)	1 (2.4)	2 (4.4)	1 (2.3)	0.7	2 (4.7)	0 (0.0)	2 (5.1)	3 (6.3)	0.77
Charlson comorbidity index	4.0 (2.0-5.0)	4.0 (3.0-6.0)	4.0 (2.8-5.0)	4.0 (2.0-4.0)	2.0 (2.0-4.0)	<0.01	4.0 (3.0-5.0)	4.0 (2.0-4.0)	4.0(2.0-5.0)	3.0 (2.0-4.0)	0.02
Frailty componets											
Weight loss (n, (%))	5 (2.9)	2 (4.7)	0 (0)	1 (2.2)	2 (4.7)	0.85	2 (4.7)	1 (2.3)	1 (2.6)	1 (2.1)	0.51
Exhaustion (n, (%))	28 (16.2)	10 (23.3)	5 (11.9)	7 (15.6)	6 (14.0)	0.33	12 (27.9)	7 (16.3)	3 (7.7)	6 (12.5)	0.03
Low physical activity (n, (%))	23 (13.3)	7 (16.3)	5 (11.9)	5 (11.1)	6 (14.0)	0.73	6 (14.0)	4 (9.3)	7 (17.9)	6 (12.5)	0.88
Gait speed, men (m/s)	1.0±0.3	0.72 (0.55-0.78)	0.89 (0.87- 0.93)	1.06 (1.02-1.10)	1.23 (1.19-1.31)	<0.01	0.85 (0.70-0.93)	0.97 (0.80-1.07)	0.94 (0.86-1.11)	1.18 (1.02-1.25)	<0.01
Gait speed, women (m/s)	0.9±0.2	0.63 (0.53-0.71)	0.89 (0.85-0.90)	1.00 (0.94-1.07)	1.24 (1.15-1.29)	<0.01	0.87 (0.62-1.02)	0.87 (0.62-1.16)	0.92 (0.88-1.10)	1.03 (0.93-1.23)	0.11
Handgrip strength, men (kg)	27.2±7.1	22.6 (20.1- 26.3)	23.9 (21.0-29.9)	28.9 (23.9-28.9)	32.9 (24.9-35.9)	<0.01	20.1 (17.4-20.7)	23.9 (23.1-24.9)	28.8 (26.9-30.9)	35.5 (33.3-38.0)	<0.01
Handgrip strength, women (kg)	17.7±4.6	15.6 (10.9-18.6)	17.2 (15.1-20.4)	20.0 (13.4-20.0)	18.9 (16.2-21.9)	0.15	12.7(10.6-14.5)	16.1 (15.2-16.3)	19.2 (17.2-19.9)	21.9 (21.5-23.1)	<0.01

We observed a significant GS quartile-dependent difference in age (P < 0.01), body mass index (BMI) (*P* = 0.03), serum creatinine level (*P* < 0.01), single-pool Kt/V (*P* = 0.01), dementia (*P* = 0.02), Charlson comorbidity index (CCI) (*P* < 0.01) [20], the presence of diabetes (*P* < 0.01), history of CVD (*P* < 0.01), and hemiplegia (*P* < 0.01). HGS showed a significant quartile-dependent difference in age (*P* < 0.01), BMI (*P* = 0.02), serum albumin (*P* < 0.01), creatinine (*P* < 0.01), calcium (*P* = 0.03), the presence of dementia (*P* = 0.02), CCI (P = 0.02), exhaustion (*P* = 0.03). The presence of diabetes and a history of CVD showed no significant difference across the four HGS quartiles.

**Fig. 2:**
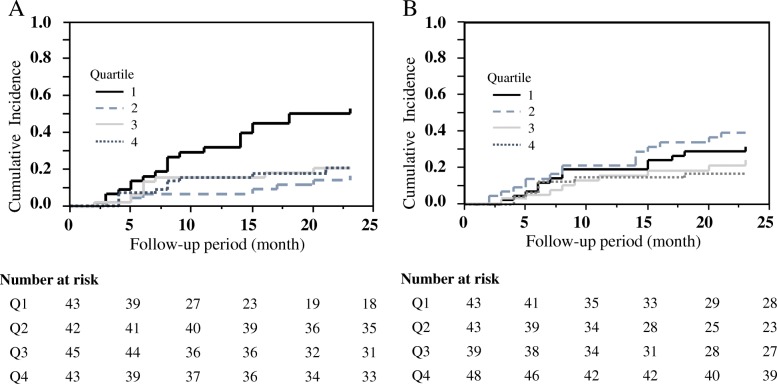
Cumulative incidence of cardiovascular events based on sex-specific quartiles of GS (**a**) and HGS (**b**). The sex-specific cut-offs for GS were < 0.82 m/s, 0.82–0.95 m/s, 0.95–1.15 m/s, and > 1.15 m/s in men, and < 0.81 m/s, 0.81–0.93 m/s, 0.93–1.11 m/s, and > 1.11 m/s in women. The sex-specific cut-offs for HGS were < 22.1 kg, 22.1–26.2 kg, 26.2–32.1 kg, and > 32.1 kg in men, and < 15.0 kg, 15.0–17.1 kg, 17.1–21.6 kg, and > 21.6 kg in women

During the 2-year follow-up period, CV events occurred in 46 patients (26.6%): five (3.0%) had CV-related death, 12 (6.9%) experienced MI and angina, 11 (6.4%) experienced stroke, eight (4.6%) were hospitalized for congestive heart failure, and ten (5.8%) experienced PAD. The incidence rate of total CV events was 17.0 per 100 person-years at risk. The incidence rates of CV death, MI, MI and angina, stroke, congestive heart failure, and PAD were 1.7, 1.0, 5.0, 4.4, 3.4, and 4.7 per 100 person-years. The rates of MI and angina were the highest among the CV events in HD patients. Analysis according to the quartiles of GS revealed that the 2-year cumulative incidence rates were 53.1, 16.8, 20.8, and 21.0 for Q1 through to Q4, respectively. There was a significant difference in CV events across the stratified GS. Patients in the lowest quartile had a significantly higher risk of CV events than those in the higher three quartiles (log-rank test, *P* = 0.0005) (Fig. 2a). The 2-year cumulative incidence rates were 31.5, 39.3, 24.2, and 16.7 for Q1 through Q4 for HGS. There was no significant difference in CV events across various HGSs (Fig. 2b).

In the univariate Cox regression analysis, old age, diabetes, history of CVD, low creatinine generation rate, low GS, and low HGS were revealed to be significantly associated with CV events when these explanatory variables were treated as continuous variables (Table 2).

**Table 2:** Clinical and biochemical parameters affecting CV events by univariate Cox model

Factors	Hazard ratio (95% CI)	*p* value
Age (years)	1.04 (1.01–1.07)	0.01
Gender (M)	0.89 (0.48–1.71)	0.71
Dialysis duration (years)	0.98 (0.93–1.02)	0.39
Diabetes	1.87 (1.04–3.47)	0.04
History of CVD	2.34 (1.31–4.31)	< 0.01
Charlson comorbidity index	1.24 (1.04–1.46)	0.02
%CGR	0.98 (0.97–0.99)	0.04
BMI (kg/m2)	1.02 (0.95–1.10)	0.55
GNRI^a^	1.06 (0.51–2.01)	0.87
Haemoglobin (g/dL)	1.27 (0.92–1.73)	0.14
Albumin (g/dL)	0.47 (0.16–1.31)	0.15
LDL-cholesterol (mg/dL)	1.01 (0.99–1.02)	0.36
intact PTH (pg/mL)	1.00 (1.00–1.00)	0.63
C-reactive protein (mg/dL)	1.24 (0.85–1.60)	0.22
Gait speed (m/s)	0.16 (0.05–0.54)	< 0.01
Handgrip strength (kg)	0.96 (0.92–0.99)	0.04

*CVD* cardiovascular disease, *%CGR* % creatinine generation rate, *BMI* body mass index, *GNRI* geriatric nutritional risk index

^a^GNRI = 14.89 × albumin (g/dL) + 41.7 × (body weight/ideal body weight)

**Fig. 3:**
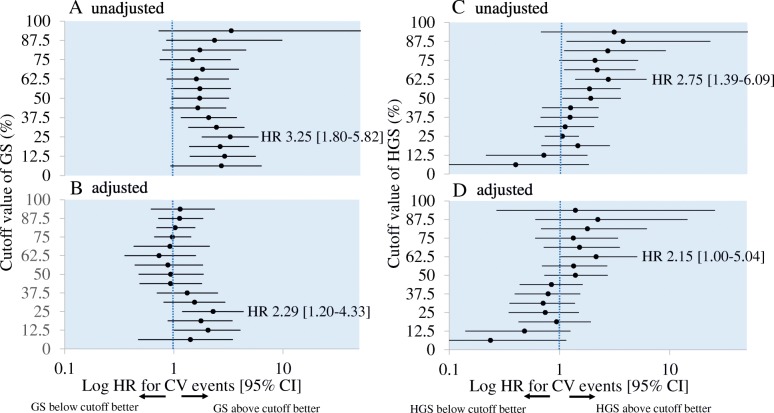
Log HRs for cardiovascular events based on 16 sex-specific quantiles of GS and HGS. Univariate (**a**) and adjusted (**b**) HRs of group whose GS is below each cut-off value vs. group whose GS is above the cut-off value. Univariate (**c**) and adjusted (**d**) HRs of group whose HGS is below each cut-off value vs. group whose HGS is above the cut-off value. Data are presented as HRs (circle) and 95% CI (bar). HRs were calculated using Cox proportional hazards models adjusted for age, sex, diabetes, and history of CVD. The cut-off values of GS quantiles were < 0.55, 0.55–0.72, 0.72–0.77, 0.77–0.82, 0.82–0.87, 0.870.89, 089–0.92, 0.92–0.96, 0.96–1.02, 1.02–1.06, 1.06–1.10, 1.10–1.15, 1.15–1.19, 1.19–1.23, 1.23–1.31, and > 1.13 m/s in men, and < 0.52, 0.52–0.62, 0.62–0.70, 0.70–0.81, 0.81–0.87, 0.87–0.89, 0.89–0.92, 0.920.93, 0.93–0.95, 0.95–1.01, 10.1–1.07, 1.07–1.12, 1.12–1.16, 1.16–1.24, 1.24–1.29, and > 1.29 m/s in women. The cut-off values of HGS quantiles were < 17.4, 17.4–20.0, 20.0–20.7, 20.7–22.2, 22.2, 23.1, 23.1–23.9, 23.9–24.9, 24.9–26.2, 26.2–26.9, 26.9–29.0, 29.0–31.0, 31.0–32.1, 32.1–33.6, 33.6–35.7, 35.7–38.6, and > 38.6 kg in men, and < 10.5, 10.5–12.6, 12.6–14.2, 14.2–15.0, 15.0–15.2, 15.2–16.1, 16.116.3, 16.3–17.1, 17.1–18.3, 18.3–19.7, 19.7–20.7, 20.7–21.6, 21.6–21.9, 21.9–22.1, 22.1–24.7, and > 24.7 kg in women

To explore the thresholds of GS and HGS for CV events, patients were classified into two groups, above and below each quantile of GS and HGS, which were further divided into 16 quantiles (Fig. 3). The unadjusted HRs for GS rose from the middle of Q1 (12.5th percentile) and became highest (HR: 3.25, 95% CI: 1.22–2.67; *P* < 0.01) at 0.82 m/s in men and 0.81 m/s in women between Q1 and Q2 (25th percentile) (Fig. 3a). The unadjusted HRs of HGS peaked at the middle of Q3 (62.5th percentile) (HR: 3.76, 95% CI: 1.16–23.0; *P* = 0.02, at 29.0 kg in men and 19.7 kg in women) and the second peak was seen in the middle of Q4 (87.5th percentile) (HR: 3.76, 95% CI: 1.16–23.0; P = 0.02, at 35.7 kg in men and 22.1 kg in women) (Fig. 3c). After adjustment for age, sex, dialysis vintage, diabetes, and history of CVD, both GS and HGS were found to be independently associated with CV events. Participants in the <25th percentile of GS (Fig. 3b), and those in the < 62.5th percentile of HGS (Fig. 3d) exhibited a HR of 2.29 (95% CI: 1.20–4.33; *P* = 0.01), and 2.15 (95% CI: 1.00–5.04; *P* < 0.05) for CV events compared with the remaining participants. These thresholding quantile points of unadjusted and adjusted HRs were consistent; the GS threshold was 0.82 m/s in men and 0.81 m/s in women, while that of HGS was 29.0 kg in men and 19.7 kg in women.

## Discussion

In this study, among Japanese HD outpatients who were able to walk independently, slow usual GS and weak HGS were significantly associated with CV events, independent of age, sex, HD duration, and medical history. When referring to the incidence rate of CV events between HD and non-HD patients in the previous studies (Additional file 1: Table S1), the incidence rate of MI and stroke in HD patients are higher than those of the general population.

The cut-off points of GS were identified as 0.82 m/s in men and 0.81 m/s in women, and those of HGS were 29.0 kg in men and 19.7 kg in women; values below these cut-offs indicated a significant association with CV events. The proportion of patients below the cut-off for GS was small, while that of patients below the cut-off for HGS was more than half. This is the first study to indicate the cut-off points of usual GS and HGS for CV events in patients with ESRD undergoing HD.

The threshold of GS for CV events could vary owing to subjects’ background and studies’ endpoints. Fatal CV was associated with GS < 0.8 m/s at a mean age of 76 years in Iceland [21], or < 1.3 m/s for men and < 1.1 m/s for women in general civil servants in London [22]. The Iceland cohort were aged average 76 and 6 m walk test whereas those in London were 61 i.e. more than a decade younger and 2.44 m walk test. The threshold of GS has been revealed to be < 0.9 m/s to affect the incidence of PAD in several studies [23–25]. In addition, a GS of < 0.8 m/s has been associated with ischaemic heart disease, heart failure, and stroke in a cross-sectional study [26, 27]. Variation in the GS threshold across previous studies might be due to the diversity of protocols, walking distances, measurement methods, and starting positions [28]. Walking distances ranged from 2.44–20 m, although the 4-m assessment has been frequently reported in the literature [14]. The cut-off points of GS in the study are similar to the cut-off points 0.8 m/s for defining low physical performance in sarcopenia [29].

HGS is a strong predictor of CVD. In community-dwelling populations, the multivariate-adjusted HR for CV events per 5 kg decreases in HGS was 1.21 (1.14–1.29, I^2^ = 95.5%); a linear association between HGS within 56 kg and CV events was reported to be significant [15]. For specific CV events, the multivariate-adjusted HR associated with a 5 kg HGS decrease for coronary heart disease (1.07, 1.03–1.11, I^2^ = 91.6%) and stroke (1.09, 1.05–1.14, I^2^ = 91.0%) was found to be significant. Several studies have also shown that HGS is significantly associated with CV-related death in both men and women [30–32], and with heart failure, stroke, and ischemic heart disease in men [16]. The thresholds for CV-related death were < 28 kg in men and < 17 kg in women aged > 65 years, < 47.0 kg in men and < 24.0 kg in women aged 40–64 years [33], and < 30.0 kg in Japanese men and < 16.5 in Japanese women aged > 65 years [32]. In HD patients, thresholds for such CV events have been reported to be < 30.0 kg in men and < 20.0 kg in women [34], similar to the results observed in the present study. However, protocols for HGS evaluation among studies were not consistent with respect to the arm position of measurement, choice of arm side, the reference to use [30, 32–35], and in the standing or seated position [36, 37]. In addition, fistula placement in HD may influence the HGS. HGS was reported to be significantly lower in the access-side limb than in the opposite side after arteriovenous fistula placement [38], and was directly related to thenar oxygenation and to finger systolic pressure only in the access-side extremity [39]. There is a need for the standardization of the techniques used for HGS evaluation in HD patients.

The mechanisms to explain the association of GS and HGS with CV events are not fully understood. For GS, walking was the most common and accessible mode of physical activity, and physical activity partially prevents age-related cardiac remodelling [40]. Physical activity has beneficial effects on obesity, diabetes, lipid profile, endothelial function and insulin sensitivity, and inflammation, which are all important risk factors of CV events [41]. A recent British cohort suggested that increased relative exercise intensity provides a greater stimulus for physiological adaptations known to influence CV-related mortality [41]. Similarly, the effect of low muscle strength is mediated through a reduced incidence of abdominal adiposity, weight gain, insulin resistance, metabolic syndrome, hypertension, and chronic inflammation [11, 12, 42]. A recent review reported that HGS could be an overall indicator of the integrity of the central nervous system and could reflect changes in the aging process [43].

The current study has some limitations. First, a common limitation of observational studies is that other unmeasured confounding factors of CV events are of concern. HD patients have CKD-related CV risk factors as well as traditional risk factors. We did not evaluate several CKD-related CV risks such as overload/ultrafiltration volumes, oxygen saturation and medicines e.g. opioids and sedatives. Second, this study was conducted only for the Japanese population in Japan, and there are possibilities for the different degrees of impact of GS and HGS from other ethnicities or other circumstances of HD. However, aging of HD patients and increasing frailty and sarcopenia is expected to become a worldwide issue [2, 4, 44].

Finally, more interventional studies on physical performance measures should be performed to elucidate whether an improvement in GS and/or HGS above the thresholds could reduce the incidence of CV events.

## Conclusions

We conclude that poor walking performance (men < 0.82 m/s, women < 0.81 m/s) and weak HGS (men < 29.0 kg, women < 19.7 kg) are suggested to be increased CV events in HD patients without physical disability. The walking test and grip force test are inexpensive and relatively simple to measure for the cardiovascular assessment of HD patients in daily practice.

## Additional file

Additional file 1: **Table S1.** Comparison of the incidence of cardiovascular disease among cohorts in the United States and Japan. (DOCX 15 kb)

## Abbreviations

BMI: Body mass index
CCI: Charlson comorbidity index
CI: Confidence interval
CKD: Chronic kidney disease
CT: Computed tomography
CV: Cardiovascular
CVD: Cardiovascular disease
ESRD: End-stage renal disease
GS: Gait speed
HbA1c: Haemoglobin A1c
HD: Haemodialysis
HGS: Handgrip strength
HR: Hazard ratio
MI: Myocardial infraction
MRI: Magnetic response imaging
PAD: Peripheral artery disease
