# Supplementary material for: Association of gait speed and grip strength with risk of cardiovascular events in patients on haemodialysis: a prospective study
Source: BMC Nephrol. 2019 May 30;20:196. doi: 10.1186/s12882-019-1370-6 (PMC6543665; doi:10.1186/s12882-019-1370-6)
Supplement: Supplementary file 1 — Table S1. Comparison of the incidence of cardiovascular disease among cohorts in the United States and Japan. (DOCX 15 kb) [file 12882_2019_1370_MOESM1_ESM.docx]

| **Additional Table S1. Comparison of the incidence of cardiovascular disease among cohorts in the United States and Japan.** | | | | | | |
| --- | --- | --- | --- | --- | --- | --- |
|  |  |  |  |  |  |  |
| **study** | **subjects** | **country** | **Rate (per 1000 person -years)** | | | |
|  |  |  | **CV events** | **MI** | **Stroke** | **CHF** |
| CKD-JAC^1^ | CKD patients  (eGRF 10-59 mi/min/1.73m^2^) | Japan | 23 | 1.6 | 5.3 | 8.6 |
| CRIC^2^ | CKD patients  (eGRF 20-70 mi/min/1.73m^2^) | United States | 38 | 13 | 6 | 26 |
| OKIDS^3^ | Dialysis patients | Japan |  |  | 17.6 |  |
| Framingham (men/women)^4,5^ | General population | United States |  | 7.1/4.2 | 2.5/1.9 |  |
| JALS (men/women)^6^ | General population | Japan |  | 1.2/0.5 | 4.4/2.9 |  |
| CKD-JAC: Chronic Kidney Disease Japan Cohort, CRIC: Chronic Renal Insufficiency Cohort, OKIDS: Okinawa Dialysis Study, JALS: Japan Arteriosclerosis, Longitudinal Study.  MI: Myocardial infarction, CHF: chronic heart failure. | | | | | | |

References

1. Tanaka K, Watanabe T, Takeuchi A, Ohashi Y, Nitta K, Akizawa T et al. Cardiovascular events and death in Japanese patients with chronic kidney disease. Kidney Int. 2017;91:227-234.

2. Denker M, Boyle S, Anderson AH, Appel LJ, Chen J, Fink JC et al. Chronic Renal Insufficiency Cohort Study (CRIC): Overview and Summary of Selected Findings. Clin J Am Soc Nephrol. 2015;10:2073-83.

3. Iseki K, Fukiyama K. Predictors of stroke in patients receiving chronic hemodialysis. Kidney Int. 1996;50:1672-5.

4. Lerner DJ, Kannel WB. Patterns of coronary heart disease morbidity and mortality in the sexes: a 26-year follow-up of the Framingham population. Am Heart J. 1986;111:383-90.

5. Wolf PA, D'Agostino RB, Kannel WB, Bonita R, Belanger AJ. Cigarette smoking as a risk factor for stroke. The Framingham Study. JAMA. 1988;259:1025-9.

6. Miura K, Nakagawa H, Ohashi Y, Harada A, Taguri M, Kushiro T et al. Four blood pressure indexes and the risk of stroke and myocardial infarction in Japanese men and women: a meta-analysis of 16 cohort studies. Circulation. 2009;119:1892-8.
